# The First Telegraph

**Published:** 1877-03

**Authors:** 


THE FIRST TELEGRAPH.—It is a curious fact (as the *Scientific American* points out) that the earliest system of telegraphy for signaling over long distances originated among the African negroes. It is still more remarkable that the means used were telephonic, and the signals were read by sound, and not by the eye, as in the case of the semaphore or other early signaling devices. The "elliembic," as the instrument used is termed, is still in existence, and has been in use from time immemorial in the Camerous country, on the west coast of Africa. By the sounds produced on striking it, the natives carry on conversation with great rapidity and at several miles distance. The noises are made to produce a perfect and distinct language, as intelligible to the operator as that uttered by the human voice.—*Journal of Chemistry.*

